# Chronic Nasal Catarrh

**Published:** 1881-04-20

**Authors:** 


### CHRONIC NASAL CATARRH.

My experience in the treatment of this disease, said Doctor Gustine in a paper read before the Medical Association of Iowa, has been a complete failure, in most instances, as far as producing a cure. Improvement has followed the use of the various remedies, both local and constitutional, recommended by the most reliable authorities on this subject; but I must confess that my success in this latitude has fallen of that which they have represented, especially in old and inveterate cases. I deem local remedies of paramount importance. For local applications, one of the best to commence with is a weak solution of permanganate of potash, one grain to the ounce of water. And let me here say, that I prepare the water myself, taking rain water and boiling it, to destroy as much as possible the infusoria always present in this fluid, and then strain. Much of the distilled and medicated waters in the shops is impregnated with this adulteration by long standing, and hence is unfit to be used in this affection. In mild cases withont erosions or ulcerations, the strength of the various local applications must be increased as the patient can bear them. Do not produce too much pain. Carbolic acid is one of our best remedies to allay fetor, and aid often in restoring the parts to a more healthy action.

One important point to remember is, that the membrane must first be cleansed of secretions more particularly from inspissated mucus, so that the medicinal substances may come directly in contact with the diseased surface. One of the best remedies for this purpose is a solution of common salt, half an ounce or one ounce to the quart of water, also a solution of muriate of ammonia is an excellent dissolvent. They can be administered by means of Thudicum's apparatus, or by a large syringe. When there is a thin, gleety discharge, you will find a weak solution of sulphate of copper, one-fourth to half a grain to the ounce of water, a most excellent remedy.

It will not do to continue one substance too long at a time. It is best to alternate them every two or three days, unless the improvement appears to be very decided under their use. Change of remedies is beneficial in this disease as well as in most others. We often fail from inattention to this rule.

When the maxillary sinus is involved, it will be necessary to extract one of the molar teeth and make an opening into the anthrum through which the injection can be administered. Sometimes the disease extends to the frontal sinus, and these are the most obstinate cases we have to deal with, because it is impossible to make our applications. Hypertrophy and atrophy of the nasal membrane are very unfavorable conditions, and, as a general rule, resist treatment a long time; and many cases of this character are never cured. These are the conditions which require strong caustic and ointments. Ointments are more effective here than solutions, because their effects are more permanent. It is often necessary to apply them in full strength, so as to change the diseased action of the part. Boracic acid has been highly recommended in the latter, and the iodine preparations in the former. A weak solution of corrosive sublimate, as an alterative, in hypertrophy, has been used with benefit.

There is one important point it will be well to impress upon your attention, and that is, the great improvement produced by climateric influences. A removal to a latitude where the temperature is scarcely ever below the freezing point has benefitted and cured, when the disease is not too far advanced, more persons than the most effective remedies. Corroborative of this position, to which allusion has been made, is the fact that the negroes who have emigrated from the far South to a more northern latitude are most universally attacked. I would, therefore, recommend as a great factor, a removal to such a climate as Florida, or Southern California.

